# Effects of Different Planting Densities on Photosynthesis in Maize Determined via Prompt Fluorescence, Delayed Fluorescence and P700 Signals

**DOI:** 10.3390/plants10020276

**Published:** 2021-01-31

**Authors:** Wanying Chen, Bo Jia, Junyu Chen, Yujiao Feng, Yue Li, Miantai Chen, Huanhuan Liu, Zhitong Yin

**Affiliations:** 1 Jiangsu Key Laboratory of Crop Genetics and Physiology, Co-Innovation Center for Modern Production Technology of Grain Crops, Key Laboratory of Plant Functional Genomics of the Ministry of Education, Joint International Research Laboratory of Agriculture & Agri-Product Safety of the Ministry of Education, Yangzhou University, Yangzhou 225009, China; CWY1533186140@163.com (W.C.); q1789553860@163.com (J.C.); fengyj2021@163.com (Y.F.); lywx28@163.com (Y.L.); 18805277362@163.com (M.C.); Liuhh@yzu.edu.cn (H.L.); 2 Huaiyin Institute of Agricultural Sciences of Xuhuai Region in Jiangsu, Huaian 223001, China; jiabo85@163.com

**Keywords:** DF induction and decay transient, modulated 820 nm reflection, OJIP transient, photosynthetic electron transport chain, shading

## Abstract

The mutual shading among individual field-grown maize plants resulting from high planting density inevitably reduces leaf photosynthesis, while regulating the photosynthetic transport chain has a strong impact on photosynthesis. However, the effect of high planting density on the photosynthetic electron transport chain in maize currently remains unclear. In this study, we simultaneously measured prompt chlorophyll *a* fluorescence (PF), modulated 820 nm reflection (MR) and delayed chlorophyll *a* fluorescence (DF) in order to investigate the effect of high planting density on the photosynthetic electron transport chain in two maize hybrids widely grown in China. PF transients demonstrated a gradual reduction in their signal amplitude with increasing planting density. In addition, high planting density induced positive J-step and G-bands of the PF transients, reduced the values of PF parameters PI_ABS_, RC/CS_O_, TR_O_/ABS, ET_O_/TR_O_ and RE_O_/ET_O_, and enhanced ABS/RC and N. MR kinetics showed an increase of their lowest point with increasing high planting density, and thus the values of MR parameters V_PSI_ and V_PSII-PSI_ were reduced. The shapes of DF induction and decay curves were changed by high planting density. In addition, high planting density reduced the values of DF parameters I_1_, I_2_, *L*_1_ and *L*_2_, and enhanced I_2_/I_1_. These results suggested that high planting density caused harm on multiple components of maize photosynthetic electron transport chain, including an inactivation of PSII RCs, a blocked electron transfer between Q_A_ and Q_B_, a reduction in PSI oxidation and re-reduction activities, and an impaired PSI acceptor side. Moreover, a comparison between PSII and PSI activities demonstrated the greater effect of plant density on the former.

## 1. Introduction

Maize is the most productive crop in the world and an important food and feed crop. Improving planting density is a key strategy used to achieve a high yield in maize [1,2]. However, maize is a high-stalk crop with long and wide leaves, thus high planting density inevitably causes mutual shading and the subsequent depression of photosynthesis in leaves around the ear. The photosynthetic performance of leaves around the ear is crucial for the determination of maize yield. Therefore, improving our understanding of the effects of mutual shading caused by high planting density on maize ear-leaf photosynthesis can aid in the advancement of plant density strategies for the development of dense-planting-resistant maize varieties.

During photosynthesis, green plants (including algae) simultaneously absorb light energy, convert carbon dioxide and water into energy-rich organic matter, and release oxygen [3]. The photosynthetic process generally comprises of light-induced linear electron transport and the Calvin cycle for CO_2_ fixation. Linear electron transport employs photosystem II (PSII) and photosystem I (PSI) to produce ATP and NADPH, two important chemical compounds used to fuel the Calvin cycle for CO_2_ fixation [4]. The majority of previous research applies chlorophyll content, photosynthetic rate, leaf area index and other indicators to determine the effects of different planting densities on the photosynthetic characteristics of maize. An increase in planting density has been observed to gradually reduce the relative chlorophyll content and net photosynthetic rate of maize leaves and increase leaf area index [5,6]. However, there is currently a lack of comprehensive information on the effect of close plant density on the linear electron transport of photosynthesis in maize.

Multifunctional plant efficiency analysis (M-PEA) has recently become a popular tool for the investigation of photosynthetic linear electron transport. M-PEA can simultaneously measure prompt chlorophyll *a* fluorescence (PF), delayed chlorophyll *a* fluorescence (DF) and modulated 820 nm reflection (MR). The kinetics of PF and delayed chlorophyll *a* fluorescence (DF) directly depend on the redox state of the PSII reaction center (P680), while those of MR are a function of the redox state of the PSI reaction center (P700). As PSI and PSII work coordinately and dynamically with a number of other electron carriers in the photosynthetic electron transport chain, fluctuations in any component of the electron chain can directly or indirectly alter the kinetics of PF, DF and MR [7]. Therefore, the three signals measured by the M-PEA provide parallel and complementary information on the entire photosynthetic linear electron transport chain, including the PSII donor side, the electron transfer between PSII and PSI and the PSI acceptor side.

We hypothesized that high planting density may affect one or multiple components of the photosynthetic linear electron chain. In the current study, we employed M-PEA to simultaneously measure the PF, DF and MR signals of Zhengdan958 and Xianyu335, the two most widely planted hybrids in China. The purpose of the study was to investigate the effect of increased planting density on the photosynthetic electron transport chain of maize and to analyze which components of the photosynthetic electron transport chain is more sensitive to increased density. The results will provide new information on the effect of high planting density on maize photosynthesis, with a direct focus on the photosynthetic electron transport chain.

## 2. Results

### 2.1. Effect of Planting Density on Yield

The ANOVA results demonstrated the significant effect of plant density on yield in both years (Table 1). In addition, the interaction between hybrid and planting density was observed to show significant effect on yield in 2020, but not in 2019. The yield of the two maize hybrids first gradually increased from the lowest level at D1 planting density to a maximum at D3 planting density, and then decreased to a relative lower level at D4 planting density.

### 2.2. Effect of Planting Density on the Net Photosynthetic Rate

**Figure 1:**
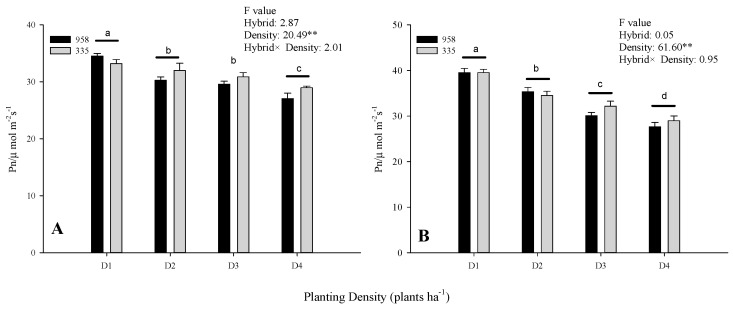
The net photosynthetic rate (*Pn*) of the two maize hybrids under different planting densities in (**A**) 2019 and (**B**) 2020. 958 denotes Zhengdan958 and 335 denotes Xianyu335. D1: 45,000 plants ha^−1^; D2: 67,500 plants ha^−1^; D3: 90,000 plants ha^−1^; D4: 112,500 plants ha^−1^. Values were presented as the means of two replicates ± standard error (SE). Different letters above the bars indicate significant differences between different densities at the 0.05 level. ** indicate significant differences at the 0.05 and 0.01 levels, respectively.

The net photosynthetic rate (*Pn*) was significantly affected by planting density in both years, but not by hybrid and the interaction between hybrid and planting density (Figure 1). *Pn* values were observed to gradually decrease with increasing planting density across hybrids and years (Figure 1). In particular, the *Pn* of Zhengdan958 in 2019 decreased by 12.33%, 14.39% and 21.71% with the increase in density compared to *Pn* D1 levels. The equivalent reductions for Xianyu335 were 3.61%, 6.99% and 12.71%, respectively. In 2020, these values were 10.60%, 23.89% and 29.99% for Zhengdan958, and 12.8%, 18.64% and 26.71% for Xianyu335, respectively.

### 2.3. Prompt Fluorescence OJIP Transient Analysis

**Figure 2:**
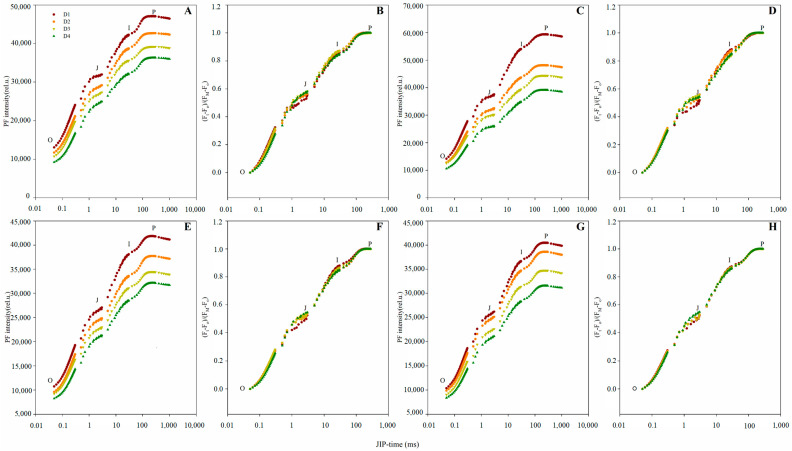
Prompt chlorophyll *a* fluorescence (PF) transients of the two maize hybrids under different planting densities. (**A**–**D**): 2019 data; (**E**–**H**): 2020 data. (A,E): Absolute values of Zhengdan958. (**B**,**F**): Normalized transients of Zhengdan958, expressed as V_t_ = [(F_t_ − F_O_)/(F_P_ − F_O_)]. (**C**,**G**): Absolute values of Xianyu335. (**D**,**H**): Normalized transients of Xianyu335, expressed as V_t_ = [(F_t_ − F_O_)/(F_P_ − F_O_)]. Signals are plotted on a logarithmic time scale.

Zhengdan958 and Xianyu335 both exhibited points O, J, I and P, presenting a typical OJIP transient (Figure 2A,C,E,G). Furthermore, points F_O_, F_J_, F_I_ and F_P_ of both hybrids gradually decreased with increasing planting density (Figure 2A,C,E,G). The O–P standardization of the OJIP transients revealed the modified shape of several OJIP transient phases following increasing planting density for both hybrids, particularly at the J-step (Figure 2B,D,F,H).

**Figure 3:**
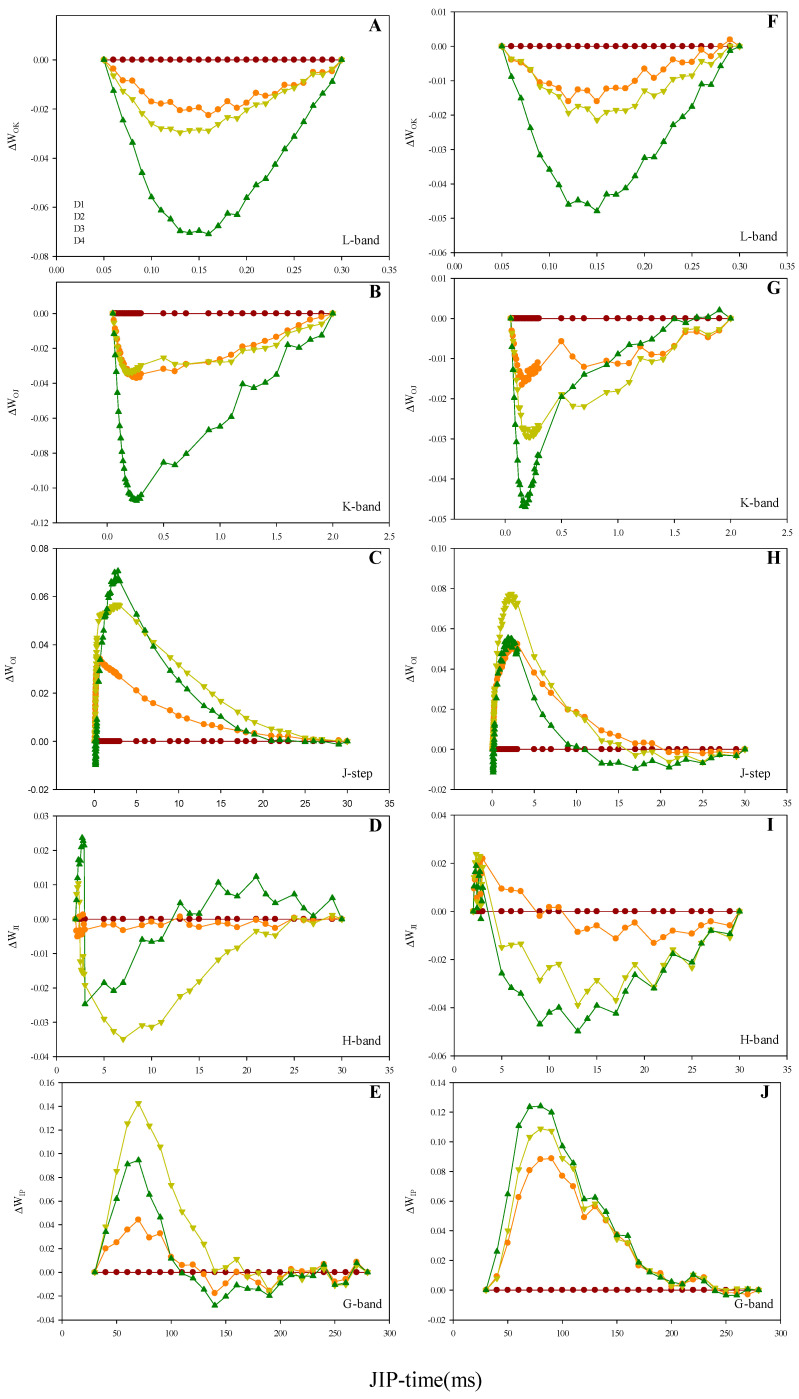
Effect of different planting densities on the shape of the OK, OJ, OI, JI and IP bands in 2019. (**A**): Zhengdan958’s O-K difference kinetics (L-band), expressed as ΔW_OK_ = W_OK_-W_OK_^D1^. (**B**): Zhengdan958’s O-J difference kinetics (K-band), expressed as ΔW_OJ_ = W_OJ_ − W_OJ_^D1^. (**C**): Zhengdan958’s O-I difference kinetics (J-step), expressed as ΔW_OI_ = W_OI_ − W_OI_^D1^. (**D**): Zhengdan958’s J–I difference kinetics (H-band), expressed as ΔW_JI_ = W_JI_-W_JI_^D1^. (**E**): Zhengdan958’s I–P difference kinetics (G-band), expressed as ΔW_IP_ = W_IP_ − W_IP_^D1^. (**F**): Xianyu335’s O–K difference kinetics (L-band), expressed as ΔW_OK_ = W_OK_ − W_OK_^D1^. (**G**): Xianyu335’s O–J difference kinetics (K-band), expressed as ΔW_OJ_ = W_OJ_-W_OJ_^D1^. H: Xianyu335’s O-I difference kinetics (J-step), expressed as ΔW_OI_ = W_OI_ − W_OI_^D1^. (**I**): Xianyu335’s J–I difference kinetics (H-band), expressed as ΔW_JI_ = W_JI_ − W_JI_^D1^. (**J**): Xianyu335’s I–P difference kinetics (G-band), expressed as ΔW_IP_ = W_IP_ − W_IP_^D1^.

**Figure 4:**
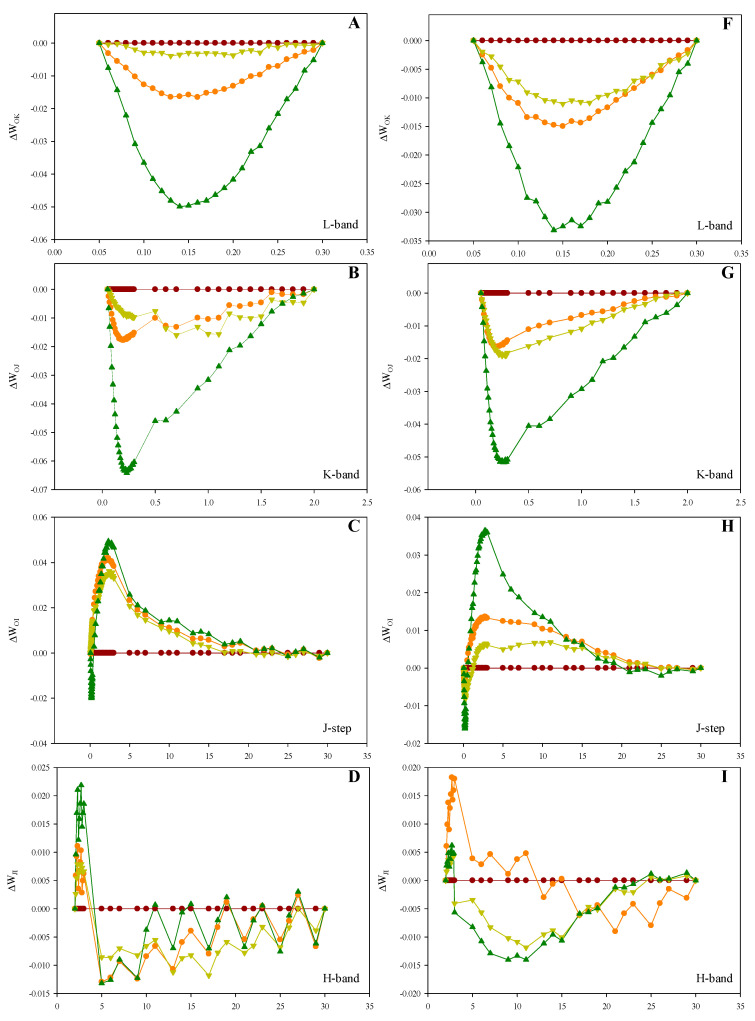

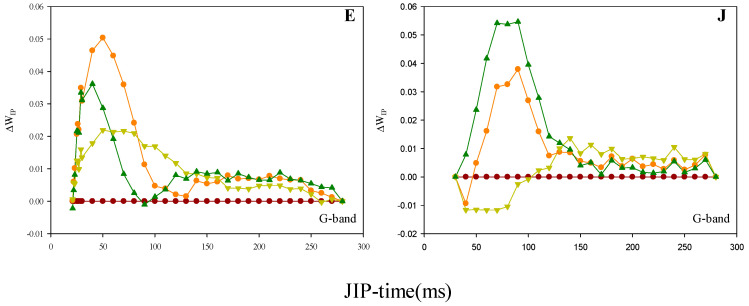
Effect of different planting densities on the shape of the OK, OJ, OI, JI and IP bands in 2020. (**A**): Zhengdan958’s O–K difference kinetics (L-band), expressed as ΔW_OK_ = W_OK_ − W_OK_^D1^. (**B**): Zhengdan958’s O–J difference kinetics (K-band), expressed as ΔW_OJ_ = W_OJ_ − W_OJ_^D1^. (**C**): Zhengdan958’s O–I difference kinetics (J-step), expressed as ΔW_OI_ = W_OI_ − W_OI_^D1^. (**D**): Zhengdan958’s J–I difference kinetics (H-band), expressed as ΔW_JI_ = W_JI_ − W_JI_^D1^. (**E**): Zhengdan958’s I–P difference kinetics (G-band), expressed as ΔW_IP_ = W_IP_ − W_IP_^D1^. (**F**): Xianyu335’s O–K difference kinetics (L-band), expressed as ΔW_OK_ = W_OK_ − W_OK_^D1^. (**G**): Xianyu335’s O–J difference kinetics (K-band), expressed as ΔW_OJ_ = W_OJ_ − W_OJ_^D1^. (**H**): Xianyu335’s O–I difference kinetics (J-step), expressed as ΔW_OI_ = W_OI_ − W_OI_^D1^. (**I**): Xianyu335’s J–I difference kinetics (H-band), expressed as ΔW_JI_ = W_JI_ − W_JI_^D1^. (**J**): Xianyu335’s I–P difference kinetics (G-band), expressed as ΔW_IP_ = W_IP_ − W_IP_^D1^.

In order to further investigate the effect of increasing planting density on each OJIP transient phase, the OJIP transients were double-normalized between F_O_ and F_K_, F_O_ and F_J_, F_O_ and F_I_, F_J_ and F_I_ and F_I_ and F_P_, respectively. The double-normalized signals of the lowest plant density (D1) were subtracted from the remaining densities to determine the ∆W_OK_, ∆W_OJ_, ∆W_OI_, ∆W_JI_ and ∆W_IP_ curves (Figure 3 and Figure 4). These curves allowed for the visualization of the L-band [8], K-band [9] J-step [10,11], H-band [12] and G-band [11], respectively. There was no sign of positive L-, K- and H-bands for the two maize hybrids under high planting density (Figure 3A,F, Figure 4A,F; Figure 3B,G, Figure 4B,G and Figure 3D,I, Figure 4D,I, respectively). A significant elevation of the J-step was associated with a high planting density in the two maize hybrids (Figure 3C,H and Figure 4C,H), while both hybrids also exhibited positive G-bands (Figure 3E,J and Figure 4E,J).

**Figure 5:**
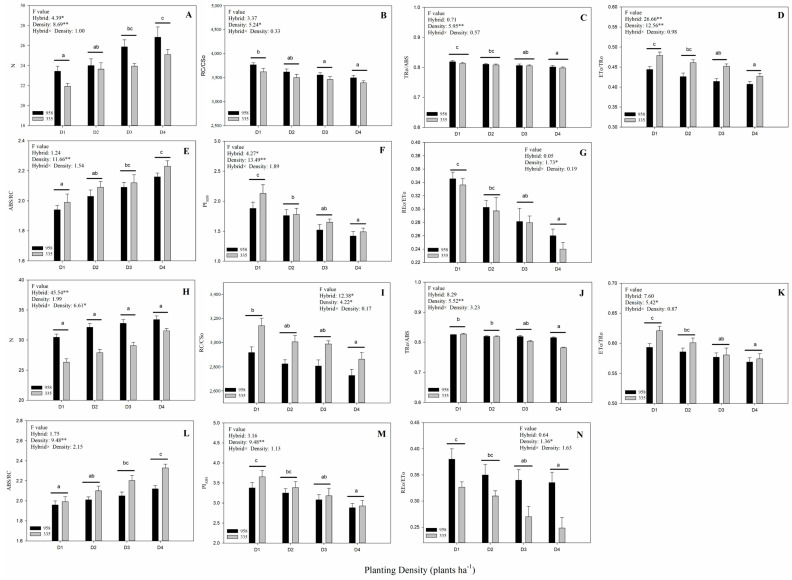
Parameters derived from PF transients under different planting densities. (**A**–**G**): 2019 data and (**H**–**N**): 2020 data. (**A**,**H**): N, the number of Q_A_ reduction events. (**B**,**I**): RC/CS_O_, the density of photosystem II (PSII) RC per unit area. (**C**,J): TR_O_/ABS, the ratio of captured light energy to absorbed light energy. (**D**,**K**): ET_O_/TR_O_, the efficiency of electron transport at Q_A-_. (**E**,**L**): ABS/RC, the light energy absorbed by the unit reaction center. (**F**,**M**): PI_ABS_, performance index (potential) for energy conservation from photons absorbed by PSII to the reduction of intersystem electron acceptors. (**G**,**N**): RE_O_/ET_O_, the efficiency of an electron beyond Q_A-_ reduced photosystem I (PSI) acceptors. Different letters (a, b, c) above the bars indicate significant differences between different planting densities at the 0.05 level. *, ** indicate significant differences at the 0.05 and 0.01 levels, respectively.

In order to quantitatively analyze the impact of increasing planting density on the photosynthetic electron transport chain, several parameters were derived from the OJIP transient using the JIP-test (Table S2) [13]. Planting density showed a significant effect on all JIP-test parameters, except the parameter N in 2020 (Figure 5). In contrast, hybrid and the interaction between hybrid and planting density showed no significant effect on most of these parameters. The values of PI_ABS_, RC/CS_O_, TR_O_/ABS, ET_O_/TR_O_ and RE_O_/ET_O_ were observed to decrease with increasing plant density, while ABS/RC and N increased significantly (Figure 5).

### 2.4. MR/MR_O_ Transient Analysis

**Figure 6:**
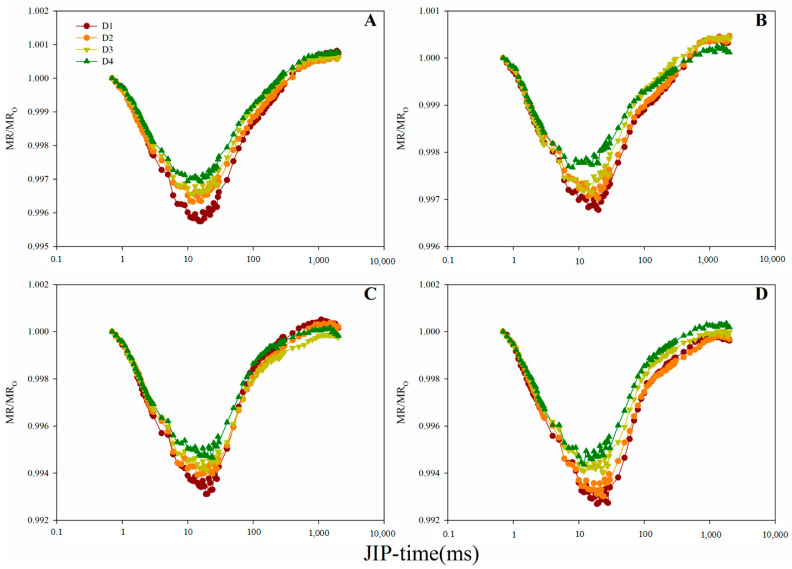
Modulated 820 nm reflection kinetics of the two maize hybrids under different planting densities. (**A**–**B**): 2019 data and (**C**–**D**): 2020 data. (**A**,**C**): Normalized values of Zhengdan958 expressed as modulated 820 nm reflection (MR)/MR_O_. (**B**,**D**): Normalized values of Xianyu335 expressed as MR/MR_O_. Signals are plotted on a logarithmic time scale. MR_O_ is the first reliable MR measurement (taken at 0.7 ms).

The lowest point of the MR/MR_O_ transient increased with planting density for both hybrids. This consequently altered the shapes of the positive and negative slopes of the MR/MR_O_ transient (Figure 6). In order to quantitatively analyze the redox variations of PSI under different planting densities, the maximum decline rate V_PSI_ and maximum rise rate V_PSII-PSI_ were calculated based on the MR/MR_O_ transient (Table 2). V_PSI_ and V_PSII-PSI_ values exhibited a gradual decrease with increasing planting density for both hybrids (Table 2).

### 2.5. DF Induction and Decay Transient Analysis

**Figure 7:**
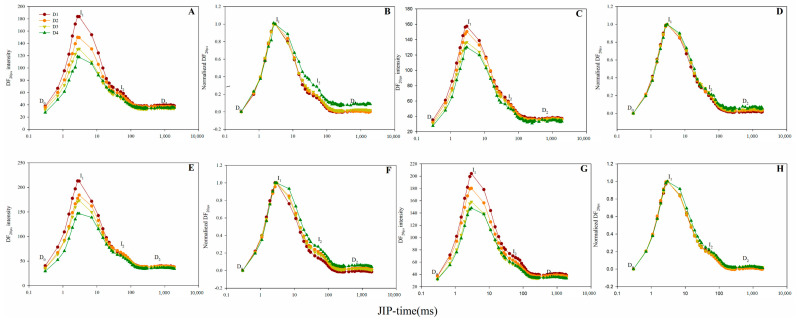
Delayed chlorophyll *a* fluorescence (DF) induction kinetics of the two maize hybrids under different planting densities. (**A**–**D**): 2019 data and (**E**–**H**): 2020 data. (**A**,**E**): Absolute values of Zhengdan958. (**B**,**F**): Normalized values of Zhengdan958, expressed as (DF_t_ − D_O_)/(DF_I1_ − D_O_). (**C**,**G**): Absolute values of Xianyu335. (**D**,**H**): Normalized values of Xianyu335, expressed as (DF_t_-D_O_)/(DF_I1_-D_O_). Signals are plotted on a logarithmic time scale. I_1_, I_2_, D_O_ and D_2_ denote the 3 ms and 100 ms peaks, the initial minimum and final plateau, respectively.

The 20-μs delay-time DF signals determined for each dark interval were used to derive the DF induction curve. The DF induction curve of the two maize hybrids exhibited an increase from an initial minimum (D_O_) to a maximum (I_1_), followed by a decrease to a plateau (D_2_) until a second maximum (I_2_) was reached (Figure 7). The DF induction curve amplitudes were observed to decrease with increasing planting density, with I_1_ exhibiting the greatest reduction (Figure 7A,C,E,G). The standardization of points D_O_ and I_1_ revealed the ability of a higher planting density to enhance I_2_/I_1_ values (Figure 7B,D,F,H).

The DF signals measured in each dark interval exhibited a polyphasic decreasing trend with time. The DF decay parameters *L*_1_ and *L*_2_ were calculated using the DF decay curve determined for the dark interval of the JIP-time of DF induction transient I_1_ step (Figure S1). These decay parameters represent the amounts of the redox states ZP_680_^+^ Q_A_^-^ and Z^+^P_680_Q_A_^-^Q_B_, respectively [13,14]. Results indicate that the increase in planting density induced a gradual decrease in both *L*_1_ and *L*_2_ (Table 3).

### 2.6. Correlation Analysis between Photosynthetic Parameters

Significant correlations were observed among the photosynthetic, *Pn*, JIP-test, DF and MR parameters (Table 4). For example, a significant positive correlation was observed for the following parameter pairs: *Pn* and PI_ABS_, *Pn* and ET_O_/TR_O_, *Pn* and *L*_2_, RC/CS_O_ and V_PSI_, RC/CS_O_ and V_PSII-PSI_, TR_O_/ABS and *L*_2_, RE_O_/ET_O_ and *L*_2_ (both years); *Pn* and TR_O_/ABS, *Pn* and RE_O_/ET_O_, PI_ABS_ and *L*_2_, RC/CS_O_ and *L*_1_, RC/CS_O_ and *L*_2_, TR_O_/ABS and V_PSI_, TR_O_/ABS and *L*_1_, RE_O_/ET_O_ and *L*_1_ (2019) and *Pn* and V_PSI_, *Pn* and V_PSII-PSI_, *Pn* and *L*_1_, PI_ABS_ and V_PSI_, PI_ABS_ and V_PSII-PSI_, ET_O_/TR_O_ and V_PSI_, ET_O_/TR_O_ and V_PSII-PSI_ (2020). A significant negative correlation was observed between *Pn* and ABS/RC, ABS/RC and *L*_2_ (both years); *Pn* and N, ABS/RC and *L*_1_ (2019) and N and V_PSI_, N and V_PSII-PSI_ (2020). Furthermore, V_PSI_ and V_PSII-PSI_ were both positively correlated with *L*_1_ (both years) and *L*_2_ (2019).

A significant negative correlation was observed for parameter pairs PI_ABS_ and N, N and ET_O_/TR_O_, TR_O_/ABS and ABS/RC, ABS/RC and RE_O_/ET_O_ (both years); PI_ABS_ and ABS/RC, N and RE_O_/ET_O_, RC/CS_O_ and ABS/RC (2019) and N and RC/CS_O_ (2020). A significant positive correlation was observed for PI_ABS_ and ET_O_/TR_O_, TR_O_/ABS and RE_O_/ET_O_ (both years); PI_ABS_ and TR_O_/ABS, PI_ABS_ and RE_O_/ET_O_, N and ABS/RC, RC/CS_O_ and TR_O_/ABS, RC/CS_O_ and RE_O_/ET_O_ (2019) and PI_ABS_ and RC/CS_O_, RC/CS_O_ and ET_O_/TR_O_ (2020). A positive correlation was observed between V_PSI_ and V_PSII-PSI_ and *L*_1_ and *L*_2_ for both years. 

## 3. Discussion 

The grain yields of Xianyu335 and Zhengdan958 demonstrated similar responses to changes in the planting density. In particular, the yields exhibited an initial increase from D1 to D3 and subsequently declined to minimum levels at D4, with the reduction in yields almost equal in the two hybrids (Table 1). This suggests that Xianyu335 and Zhengdan958 have a similar tolerance to high planting density. Similar results were also observed in previous studies [15]. Furthermore, Xianyu335 and Zhengdan958 also exhibited similar trends in photosynthetic variations for increasing plant density. More specifically, the enhanced mutual shading of the ear leaves resulting from the high planting density led to a continuous reduction of the photosynthetic rate for both hybrids from D1 to D4 (Figure 1). The maximum grain yield of Xianyu335 and Zhengdan958 was achieved at D3 (Table 1), and is thus regarded as a suitable planting density for both hybrids. The rise in grain yield from D1 to D3 can be attributed to the increased planting density, while the subsequent decline in grain yield following D3 is linked to the reduced photosynthesis.

Photosynthesis is a complicated multicomponent process. The damage or weakening of any of the involved component can consequently reduce photosynthesis. In this study, several photosynthetic electron transport chain related parameters were observed to be significantly correlated with photosynthetic rate (Table 4). Furthermore, the variations in the shape of the OJIP transients, DF induction and decay transients and MR kinetics of Xianyu335 and Zhengdan958 were observed following the increasing planting density (Figure 2, Figure 6 and Figure 7). This indicates the ability of a high planting density to alter the activity of the photosynthetic electron transport chain. This may subsequently play a role in the reduced photosynthesis. Therefore, we further investigated the effect of high planting density on the photosynthetic electron transport chain.

The PF OJIP transient and corresponding JIP-test parameters provide information on the electron transfer and related events occurring in the photosynthetic electron transport chain [6,16]. The JIP-test parameters RC/CS_O_ and ABS/RC, which represent the RC density per unit area and the light energy absorbed per RC, were observed to increase (Figure 5B,I) and decrease (Figure 5E,L), respectively. This suggests that the high planting density inactivated PSII RC and reduced the number of active PSII RC [17,18]. This subsequently enhanced the number of RC turnovers for a reduction of PQ pool, which can be observed by the increase in parameter N (Figure 5A,H). The JIP-test parameter TR_O_/ABS reflects the average maximum primary photochemistry quantum yield of active and inactive RCs [19,20]. The decrease in TR_O_/ABS observed under a high planting density (Figure 5C,J) may also be linked to the lower number of active RCs [21]. In addition, the overall signal strength of the OJIP transient under a high planting density may have decreased with the number of active PSII RCs (Figure 2). The OJIP transient J-step is associated with the electron flow from Q_A_ to Q_B_, whereby the higher the J-step, the greater the electron blockage at this site [10,11]. We observed the J step to increase with planting density (Figure 3C,H and Figure 4C,H), suggesting that a high planting density induced the electron transfer blockage from Q_A_ to Q_B_. This is also implied by the decrease in JIP-test parameter ET_O_/TR_O_ (Figure 5D,K), which denotes the probability of a trapped exciton moving an electron into the electron transport chain beyond Q_A_^-^. The G-band reflects the reduction of the PSI acceptor side via the electrons expelled from the PQ pool [22]. Electron-traffic jams caused by the instantaneous blockage of the PSI acceptor side can result in a positive G-band [23]. In the current study, Zhengdan958 and Xianyu335 both exhibited positive G-bands with increasing planting density (Figure 3E,J and Figure 4E,J). This indicates that high planting density decreased the functionality of the PSI acceptor side and blocked the electron transfer at this site. This is in agreement with the lower observed JIP-test parameter RE_O_/ET_O_ (Figure 4N and Figure 5G), which refers to the probability that an electron beyond Q_A_^−^ reduces an end acceptor at the PSI electron acceptor site. The JIP-test parameter PI_ABS_ integrates the information of three independent parameters (ABS/RC, TR_O_/ABS and ET_O_/TR_O_) to reflect PSII activity more accurately compared to each individual parameter [19,24,25]. The lower number of active PSII RCs and the enhanced electron transfer blockage from Q_A_ to Q_B_ reduced PI_ABS_ under a high planting density (Figure 5F,M).

The L-band of the OJIP transient is associated with the connectivity between independent PSII units, with a positive L-band suggesting a decrease in the connectivity between PSII units [26,27]. Xianyu335 and Zhengdan958 did not exhibit a positive L-band (Figure 3A,F and Figure 4A,F), suggesting that the high planting density employed in this study did not result in any impairment to the energetic connectivity of the PSII units. The K-band is linked to the oxygen-evolving-complex (OEC) at the PSII donor side, with a positive K-band suggesting the inactivation of the OEC [28,29,30]. Numerous studies have demonstrated the induction of a positive and pronounced K-band from severe abiotic stresses (e.g., drought stress, salt stress and high temperature) [19,31,32]. Our results failed to reveal a positive K-band for Xianyu335 and Zhengdan958 with increasing planting density (Figure 3B,G and Figure 4B,G). This suggests the lack of OEC damage and electron transfer capacity impairment on the PSII donor side following the increased planting density. The H-band reflects the redox process of the PQ pool, during which the electrons transferred from the PSII begin to reduce the PQ pool [33]. We did not observe a positive H-band for Xianyu335 and Zhengdan958 with increasing planting density (Figure 3D,I and Figure 4D,I), suggesting that the high planting density employed in this study did not affect the equilibrium between the oxidation and PQ pool reduction.

The decreasing and increasing slopes of the MR/MR_O_ curve reflect the oxidation and rereduction of PSI, respectively [34]. In the present study, both the slopes of the MR/MR_O_ curve were changed by high planting density (Figure 6), suggesting the marked effect of high planting density on the PSI oxidation and rereduction activities. The decrease in V_PSI_ and V_PSII-PSI_ (Table 2) indicates that high planting reduced PSI oxidation and PSI rereduction activities, respectively [8]. A PSI rereduction activity can be attributed to a lower PSII capacity to pump electrons to PSI, an increase in the relative activity of PSI compared to PSII, and/or a decrease in functionality of at PSI acceptor side. V_PSII-PSI_ exhibited a stronger correlation with V_PSI_ compared to other parameters (Table 4), suggesting that the variations in PSI relative activity may have a marked influence on the rereduction of PSI.

Delayed chlorophyll *a* fluorescence is a result of the repopulation of excited PSII antenna chlorophyll by backward electrons arriving at the active PSII RCs [35]. The intensity of the DF induction transient was lowered as the planting density increased (Figure 7A,C,E,G), suggesting that the number of active PSII RCs decreased under a high planting density. The I_1_ amplitude of the DF induction curve is related to the electron transfer capacity of the PSII donor side, PSII acceptor side and/or the number of active PSII RCs [21,36]. The reduced number of active RCs and weaker electron transfer blockage between Q_A_ and Q_B_ may explain the decrease in the I_1_ amplitude of the DF induction curve under a high planting density (Figure 7A,C,E,G). Furthermore, the reduced number of active RCs and limited electron transfer between Q_A_ and Q_B_ caused by a high planting density may lower the accumulation of the two luminescent components, ZP_680_^+^ Q_A_^−^ and Z^+^P_680_Q_A_^−^Q_B_ [37,38], reflected by parameters *L*_1_ and *L*_2_, respectively. These two parameters were observed to decrease with increasing planting density (Table 3), thus agreement with the previous observation. Point I_2_ point of the DF induction curve generally coincides with the I–P phase of the OJIP transient and the increasing phase of the MR/MR_O_ curve, suggesting that point I_2_ is linked to the reduction of the PSI acceptor side [38,39]. Consistent with the aforementioned results of PF and MR, the reduction in the I_2_ amplitude (Figure 7A,C,E,G) suggests a decreasing trend for PSI reduction activity at a high planting density. The I_2_/I_1_ ratio is associated with the relative activity of PSI compared to PSII [23,40]. The increased I_2_/I_1_ observed in our results (Figure 7B,D,F,H) suggests the greater influence of high planting density in reducing PSII compared to PSI activity.

## 4. Materials and Methods

### 4.1. Plant Material Growth and Treatment 

Two maize hybrids, Zhengdan958 and Xianyu335 were planted in the experimental field of the Agricultural College of Yangzhou University, China on 20 April 2019 and 16 May 2020. We employed four planting densities of 45,000, 67,500, 90,000 and 112,500 plants ha^−^^1^ expressed by D1, D2, D3 and D4, respectively. The experiment was based on a two-factor (hybrids and planting density) randomized block design with two replications. The area of each plot was 9 m^2^, with a fixed row length of 3 m and spacing of 0.6 m, respectively. Different planting densities were achieved by adjusting plant spacing (Table S1). The two maize hybrids were grown under natural irradiance. The daily average temperature throughout the growing seasons of 2019 and 2020 was 24.93 °C and 26.72 °C, respectively (Figure S2). The daily relative humidity was 69.05% and 76.32%, respectively. The daily sunshine duration was 4.53 h and 3.53 h, respectively. The field management followed that of local standard agronomic practices.

### 4.2. Determination of Yield 

The maize plants were manually harvested on 15 August 2019 and 27 August 2020. The ear was fully dried to a constant mass, and the yield of each plot was weighed after threshing and converted into hectare yield (kg ha^−1^).

### 4.3. Determination of Net Photosynthetic Rate 

One week after maize pollination, 5 maize plants were randomly selected from the center of each plot to determine the photosynthetic rate. The photosynthetic rate was measured using a CIRAS-3 portable gas exchange system (PP-Systems, USA). During measurements, the CIRAS-3 automatic control device-controlled CO_2_ concentration (390 µmol mol^−1^), air humidity (60%), PARi (1800 µmol m^−2^s^−1^), gas flow (100 cc/min) and leaf temperature (25 °C). Measurements were performed on the central and upper regions of ear leaf. All measurements were made in a sunny morning (9:00–11:30 AM) on the same day.

### 4.4. Prompt Chlorophyll a Fluorescence, Delayed Chlorophyll a Fluorescence and Modulated 820 nm Reflection Measurements

The multifunctional plant efficiency analyzer (M-PEA, Hansatech, Norfolk, UK) was employed to simultaneously measure the signals of PF, DF and MR. One week after maize pollination, we randomly selected 5 maize plants in the center of each plot for the measurements of these signals. A leaf disk with 20 cm length and full width was cut from the middle part of the ear leaf of each plant, and maintained in wet gauze for the dark adaptation for more than half to achieve a full dark-adapted state an hour prior to the measurements. Under the full dark-adapted state, all PSII RCs in the leaf are open, and the PF signal at the onset of illumination is F_O_. The measurement was made two times at two different positions of each leaf sample. During the measurements, an actinic light source with an intensity of 5000 µmol (photons) m^−2^s^−1^ uniformly illuminated the leaf sample surface. The PF and DF signals were measured when the actinic light was on (light interval) and off (dark interval), respectively. The first reliable MR measurement was at 0.7 ms after the first switching on of the actinic light, and the signal recorded at this time was taken as MR_O_. All measurements were made in a laboratory at room temperature (26 °C), with a 60% relative humidity.

Seven PF parameters including N, RC/CS_O_, TR_O_/ABS, ET_O_/TR_O_, ABS/RC, PI_ABS_ and RE_O_/ET_O_ were derived from the PF OJIP transient according to a JIP-test method [8]. Two MR parameters, V_PSI_ and V_PSII-PSI_, were derived from the MR/MR_O_ transient according to a previously described method [7]. Three DF parameters, I_1_, I_2_ and I_2_/I_1_, were derived from the DF induction curve, where I_1_ and I_2_ denote the first and second maxima of the DF induction curve, respectively. Another five DF parameters including *L*_1_, *L*_2_, *L*_3_, τ_1_ and τ_2_ were derived from the DF decay curve according to a previously described method [7], where *L* denotes the amplitude of the emission component and τ is the lifetime of the DF component. For a detailed description of these parameters, see Table 5.

### 4.5. Statistical Analysis

All data were analyzed using SPSS 16.0 (IBM, New York, NY, USA), with the PROC MEANS procedure employed to calculate the means of the phenotypic data. The data collected from the 5 plants in the same plot were averaged to represent the value of this plot. The plot data were used in analysis of variance (ANOVA) for effect of planting density and maize hybrid. A two-factor (hybrids and planting density) ANOVA analysis was employed. Duncan’s multiple range test was performed at *p* < 0.05. Values were presented as the means of two replicates ± standard error (SE).

## 5. Conclusions

The mutual shading among individual plants resulting from high planting density inevitably reduced maize ear leaf photosynthesis. Improving maize planting density to achieve high yield works only when the yield loss caused by decreased photosynthesis did not exceed the gain of increased planting density. Photosynthetic electron transport was required for efficient photosynthetic activity. However, the effect of high planting density on the photosynthetic electron transport chain in maize currently remained unclear. In the current study, we proposed a simultaneous measurement of PF, DF and MR in order to investigate the effect of increasing planting density on maize photosynthetic electron transport chain. The increase in planting density was observed to inactivate PSII RCs, block the electron transfer between Q_A_ and Q_B_, reduce PSI oxidation and rereduction activities and decrease the functionality of the PSI acceptor side. Furthermore, a high planting density induced a greater reduction in PSII activity compared to PSI activity. In agreement with the similar tolerance to high planting density, the two maize hybrids Xianyu335 and Zhengdan958 used in the current study demonstrated similar changes in the photosynthetic electron transport chain under high planting density. Simultaneous measurement of PF, DF and MR is a rapid, accurate and non-invasive method to investigate the changes in photosynthetic electron transport. More importantly, it can provide complementary and mutually corroborated information. Future studies using two maize hybrids with contrasting tolerance to high planting density are needed to test whether this simultaneous measurement could be used to distinguish tolerant from sensitive maize hybrids.

## Figures and Tables

**Table 1:** Analysis of variance for the effect of planting density and maize hybrid on yield.

	Hybrid	Planting Density (Plants ha^−1^)	Yield (kg ha^−1^)		Hybrid	Planting Density (Plants ha^−1^)	Yield (kg ha^−1^)
2019	Zhengdan958	D1	8205a	2020	Zhengdan958	D1	8645a
		D2	10,294a			D2	12,009b
		D3	13,601b			D3	15,348c
		D4	13,147b			D4	13,487bc
	Xianyu335	D1	8456a		Xianyu335	D1	8519a
		D2	11,565b			D2	11,934b
		D3	12,923b			D3	13,477b
		D4	12,300b			D4	12,645b
	F value	Hybrid	3 × 10^−6^		F value	Hybrid	19.26 **
		Density	27.29 **			Density	52.21 **
		Hybrid × Density	1.59			Hybrid × Density	4.12 **

Note: Different letters (a, b, c) indicate significant differences between different densities within the hybrid at the 0.05 level, ** indicate significant differences at the 0.01 levels.

**Table 2:** Parameters derived from the modulated 820 nm reflection (MR/MR_O_) of the two maize hybrids under different planting densities. V_PSI_: maximum slope decrease of MR/MR_O_; V_PSII-PSI_: maximum slope increase of MR/MR_O_; V_PSII_ = V_PSI_ + V_PSII-PSI_.

		V_PSI_	V_PSII-PSI_	V_PSII_
2019	Zhengdan958			
	D1	1.0045 ± 0.00018a	1.0055 ± 0.00022a	2.0100 ± 0.00038a
	D2	1.0039 ± 0.00018ab	1.0047 ± 0.00025ab	2.0086 ± 0.00042ab
	D3	1.0037 ± 0.00016b	1.0046 ± 0.00022b	2.0083 ± 0.00037b
	D4	1.0033 ± 0.00013b	1.0042 ± 0.00021b	2.0076 ± 0.00033b
	Xianyu335			
	D1	1.0032 ± 0.00014a	1.0037 ± 0.00015a	2.0069 ± 0.00029a
	D2	1.0029 ± 0.00009ab	1.0035 ± 0.00021a	2.0064 ± 0.00041ab
	D3	1.0028 ± 0.00014ab	1.0033 ± 0.00014a	2.0061 ± 0.00027ab
	D4	1.0023 ± 0.00018b	1.0026 ± 0.00020b	2.0049 ± 0.00037b
2020	Zhengdan958			
	D1	1.0069 ± 0.00022a	1.0075 ± 0.00025a	2.0144 ± 0.00047a
	D2	1.0061 ± 0.00026ab	1.0065 ± 0.00028ab	2.0126 ± 0.00053ab
	D3	1.0058 ± 0.00045ab	1.0059 ± 0.00041b	2.0118 ± 0.00086b
	D4	1.0053 ± 0.00025b	1.0055 ± 0.00031b	2.0109 ± 0.00055b
	Xianyu335			
	D1	1.0074 ± 0.00042a	1.0080 ± 0.00049a	2.0154 ± 0.00091a
	D2	1.0070 ± 0.00018ab	1.0078 ± 0.00020a	2.0148 ± 0.00037ab
	D3	1.0061 ± 0.00027bc	1.0069 ± 0.00022a	2.0130 ± 0.00049bc
	D4	1.0060 ± 0.00021c	1.0060 ± 0.00027b	2.0127 ± 0.00048c

Note: Different letters (a, b, c) indicate significant differences between different densities within the hybrid at the 0.05 level.

**Table 3:** DF decay parameters determined by fitting the experimental data to the time function DF (t) = *L*_1_ × exp (−t/τ_1_) + *L*_2_ × exp (−t/τ_2_) + *L*_3_, where *L*_1_, *L*_2_ and *L*_3_ are the amplitudes (in relative units) of the kinetic components, and τ_1_ and τ_2_ are their lifetimes (in ms).

		*L*_1_	*L*_2_	*L*_3_	τ_1_	τ_2_
2019	Zhengdan958					
	D1	331.93 ± 9.88a	63.67 ± 3.01a	22.17 ± 2.03a	0.02 ± 0.00a	0.28 ± 0.02a
	D2	308.33 ± 7.99ab	54.16 ± 3.06ab	19.00 ± 1.96a	0.02 ± 0.00a	0.31 ± 0.03a
	D3	282.87 ± 9.95bc	52.06 ± 3.10ab	18.74 ± 1.90a	0.02 ± 0.00a	0.29 ± 0.03a
	D4	243.60 ± 10.01c	46.17 ± 2.81b	17.27 ± 1.88a	0.02 ± 0.00a	0.27 ± 0.04a
	Xianyu335					
	D1	272.96 ± 9.83a	56.25 ± 3.08a	20.09 ± 2.03a	0.02 ± 0.00a	0.30 ± 0.00a
	D2	263.53 ± 9.90a	50.58 ± 2.85ab	18.38 ± 1.97a	0.02 ± 0.00a	0.29 ± 0.01a
	D3	242.83 ± 8.74ab	49.36 ± 3.01ab	17.67 ± 1.91a	0.02 ± 0.00a	0.30 ± 0.01a
	D4	222.63 ± 9.90b	44.36 ± 3.05b	16.84 ± 2.91a	0.02 ± 0.00a	0.29 ± 0.02a
2020	Zhengdan958					
	D1	296.34 ± 10.79a	56.26 ± 2.90a	23.81 ± 2.81a	0.02 ± 0.00a	0.30 ± 0.03a
	D2	224.09 ± 13.84b	48.82 ± 2.88ab	20.12 ± 2.73a	0.02 ± 0.00a	0.32 ± 0.01a
	D3	209.20 ± 9.90bc	46.35 ± 3.05ab	19.66 ± 3.71a	0.02 ± 0.00a	0.32 ± 0.01a
	D4	184.86 ± 9.77c	43.75 ± 2.86b	18.46 ± 2.89a	0.02 ± 0.00a	0.34 ± 0.02a
	Xianyu335					
	D1	247.05 ± 9.66a	49.93 ± 2.96a	23.81 ± 1.97a	0.02 ± 0.00a	0.28 ± 0.03a
	D2	224.38 ± 7.95ab	47.55 ± 2.83a	20.70 ± 1.88a	0.02 ± 0.00a	0.30 ± 0.02a
	D3	216.77 ± 9.98ab	43.36 ± 3.97a	19.60 ± 1.91a	0.02 ± 0.00a	0.30 ± 0.02a
	D4	206.02 ± 9.84b	41.54 ± 3.02b	18.46 ± 2.98a	0.02 ± 0.00a	0.32 ± 0.03a

Note: Different letters (a, b, c) indicate significant differences between different densities within the hybrid at the 0.05 level.

**Table 4:** Correlation analysis between photosynthetic parameters.

	N	RC/ CS_O_	TR_O_/ ABS	ET_O_/ TR_O_	ABS/RC	PI _ABS_	RE_O_/ ET_O_	V_PSI_	V_PSII-_ _PSI_	*L*_1_	*L*_2_	*Pn*
N		−0.46	−0.70	−0.92 **	0.74*	-0.94 **	−0.76 *	−0.10	−0.01	−0.37	−0.61	−0.87 **
RC/ CS_O_	−0.99 **		0.94 **	0.22	−0.86 **	0.65	0.90 **	0.91 **	0.87 **	0.94 **	0.97 **	0.70
TR_O_/ ABS	−0.24	0.22		0.52	−0.93 **	0.82*	0.99 **	0.76*	0.70	0.88 **	0.98 **	0.88 **
ET_O_/ TR_O_	-0.88 **	0.88 **	0.55		-0.58	0.84 **	0.61	0.16	0.24	0.09	0.39	0.78*
ABS/ RC	0.16	−0.17	−0.96 **	−0.53		−0.90 **	−0.96**	-0.65	−0.58	−0.77 *	−0.88 **	-0.78*
PI_ABS_	−0.84 **	0.85 **	0.64	0.97 **	-0.64		0.89 **	0.31	0.22	0.52	0.74*	0.86 **
RE_O_/ ET_O_	0.18	0.17	0.87 **	0.22	−0.92 **	0.34		0.68	0.61	0.81*	0.95 **	0.89 **
V_PSI_	−0.88 **	0.88 **	0.43	0.95 **	−0.44	0.94 **	0.17		0.99 **	0.94 **	0.82*	0.41
V_PSII-_ _PSI_	−0.91 **	0.90 **	0.47	0.91 **	−0.44	0.94 **	0.16	0.97 **		0.90 **	0.76*	0.32
*L*_1_	−0.44	0.48	0.49	0.59	−0.61	0.70	0.52	0.74 *	0.71 *		0.91 **	0.63
*L*_2_	−0.27	0.30	0.76*	0.57	−0.86 **	0.69	0.81*	0.64	0.62	0.91 **		0.84 **
*Pn*	−0.66	0.69	0.64	0.85 **	−0.71*	0.93 **	0.50	0.88 **	0.87 **	0.89 **	0.86 **	

Note: Correlation coefficients listed in lower triangle and upper triangle were determined from 2020 and 2019 data, respectively. *, ** indicate significant at the 0.05 and 0.01 levels, respectively.

**Table 5:** The PF, DF and MR parameters used in this study.

**Parameters of PF**	
N = (S_M_/S_S_) = S_M_M_O_(1/V_J_)	the number of Q_A_ reduction events
RC/CS_O_ = φPo (V_J_/M_O_) (ABS/CS_O_)	the density of PSII RC per unit area
φPo = TR_O_/ABS = [1 − (F_O_/F_M_)]	the ratio of captured light energy to absorbed light energy
ψEo = ET_O_/TR_O_ = (1 − V_J_)	the efficiency of electron transport at Q_A-_
PI_ABS_ = (RC/ABS)·[φPo/(1 − φPo)]·[ψo/(1 − ψo)]	performance index (potential) for energy conservation from photons absorbed by PSII to the reduction of intersystem electron acceptors
δRo = RE_O_/ET_O_ = (1 − V_I_)/(1 − V_J_)	the efficiency of an electron beyond Q_A-_ reduced PSI acceptors
**Parameters of DF**	
*L*_1_, *L*_2_ and *L*_3_	the amplitude of the emission component
τ_1_ and τ_2_	the lifetime of the DF component
I_1_	the first maxima of the DF induction curve
I_2_	the second maxima of the DF induction curve
I_2_/I_1_	the second maxima divided by the first maxima of the DF induction curve
**Parameters of MR**	
V_PSI_	The maximum PSI oxidation rate
V_PSII-PSI_	maximum PSI reduction rate

## Data Availability

The data presented in this study is contained within the article.

## Supplementary Materials

The following are available online at https://www.mdpi.com/2223-7747/10/2/276/s1, Figure S1: Kinetics of delayed fluorescence DF (in arbitrary units) at the characteristic steps I1 (7 ms JIP-time) of the two maize hybrids under different planting densities. (A,B): 2019 data; (C,D): 2020 data. (A,C): Absolute values of Zhengdan958. (C,D): Absolute values of Xianyu335. Figure S2: Daily mean temperature, daily relative humidity and daily sunshine duration during the growing season in (A) 2019 and (B) 2020. Table S1: Planting density of the maize in field, Table S2: Formulae and glossary of terms used by the JIP-test for Chl *a* fluorescence transient OJIP analysis.

## Abbreviations

PF	instantaneous chlorophyll fluorescence
MR	modulated 820 nm light reflection
DF	chlorophyll a delayed fluorescence
PSI	photosystem I
PSII	photosystem II
P680	PSII reaction center
P700	PSI reaction center
N	number of Q_A_ reduction events
RC/CS_O_	density of PSII RC per unit area
TR_O_/ABS	ratio of captured light energy to absorbed light energy
ET_O_/TR_O_	efficiency of electron transport at Q_A_^-^
ABS/RC	light energy absorbed by the unit reaction center
PI_ABS_	performance index (potential) for energy conservation from photons absorbed by PSII to the reduction of intersystem electron acceptors
RE_O_/ET_O_	efficiency of an electron beyond Q_A_^−^ reduces PSI acceptors
*Pn*	net photosynthetic rate
Q_A_	photosystem II primary quinone acceptor
Q_B_	photosystem II secondary quinone acceptor
PQ	plastoquinone
V_PSI_	maximum falling slope of the MR/MR_O_ curve
V_PSII -PSI_	maximum rising slope of the MR/MR_O_ curve
OEC	Oxygen-evolving complex
*L*_1_, *L*_2_ and *L*_3_	amplitudes of the DF decay kinetic components
