# Supplementary material for: Effects of Different Planting Densities on Photosynthesis in Maize Determined via Prompt Fluorescence, Delayed Fluorescence and P700 Signals
Source: Plants (Basel). 2021 Jan 31;10(2):276. doi: 10.3390/plants10020276 (PMC7910836; doi:10.3390/plants10020276)
Supplement: Supplementary file 1 [file plants-10-00276-s001.pdf]

plants

Article

Effects of different planting densities on photosynthesis in maize determined via prompt fluorescence, delayed fluorescence and P700 signals

Wanying Chen [1,+], Bo Jia [2,+], Junyu Chen [1], Yujiao Feng [1], Yue Li [1], Miantai Chen [1], Huanhuan Liu [1] and Zhitong Yin[1,*]

[1] Jiangsu Key Laboratory of Crop Genetics and Physiology/Co-Innovation Center for Modern Production Technology of Grain Crops/Key Laboratory of Plant Functional Genomics of the Ministry of Education/Joint International Research Laboratory of Agriculture & Agri-Product Safety of the Ministry of Education, Yangzhou University, Yangzhou, 225009, China; 1533186140@qq.com (W.C); 2577460773@qq.com (J.C); 952389585@qq.com (Y.F); 1032141759@qq.com (Y.L); tocmt@qq.com (M.C); Liuhh@yzu.edu.cn (H.L)
[2] Huaiyin Institute of Agricultural Sciences of Xuhuai Region in Jiangsu, Huaian 223001, China; jiabo85@163.com (B.J)
* Correspondence: ztyin@yzu.edu.cn (Z.Y)
+ Those authors contributed equally to this work

Table S1. Planting density of the maize in field.

Planting Density (plants ha^{-1})	Treatment	Row Spacing(m)	Plant Spacing(m)
45000	D1	0.6	0.43
67500	D2	0.6	0.27
90000	D3	0.6	0.2
112500	D4	0.6	0.15

Table S2. Formulae and glossary of terms used by the JIP-test for Chl a fluorescence transient OJIP analysis.

Term/Formula	
Ft	Fluorescence at time t after the onset of actinic illumination
$F_O \cong F_{20\mu s}$ or $F_{50\mu s}$	Minimal fluorescence, when all PSII RCs are open
$F_L=F_{150\mu s}$	Fluorescence intensity at the L-step (150 μs) of OJIP
$F_K=F_{300\mu s}$	Fluorescence intensity at the K-step (300 μs) of OJIP
$F_J=F_{2ms}$	Fluorescence intensity at the J-step (2 ms) of OJIP
$F_I=F_{30ms}$	Fluorescence intensity at the I-step (30 ms) of OJIP
$F_P=F_M$	Maximal recorded fluorescence intensity, at peak P of OJIP
$F_V=F_M-F_O$	Maximal variable fluorescence
$V_t=(F_t-F_O)/(F_M-F_O)$	Relative variable fluorescence at time t
$V_K=(F_K-F_O)/(F_M-F_O)$	Relative variable fluorescence at the K-step
$V_J=(F_J-F_O)/(F_M-F_O)$	Relative variable fluorescence at the J-step
$W_t=(F_t-F_O)/(F_J-F_O)$	Relative variable fluorescence FV to the amplitude F_J-F_O
$W_L=W_{150\mu s}=(F_{150\mu s}-F_O)/(F_J-F_O)$	Relative variable fluorescence at the L-step to the amplitude F_J-F_O
$W_K=W_{300\mu s}=(F_{300\mu s}-F_O)/(F_J-F_O)$	Relative variable fluorescence at the K-step to the amplitude F_J-F_O
$M_O=4(F_{300\mu s}-F_O)/(F_M-F_O)$	Approximated initial slope (in ms-1) of the fluorescence transient normalized on the maximal variable fluorescence F_V
$W_{OK}=(F_t-F_O)/(F_K-F_O)$	Ratio of variable fluorescence F_t-F_O to the amplitude F_K-F_O
$W_{OJ}=(F_t-F_O)/(F_J-F_O)$	Ratio of variable fluorescence F_t-F_O to the amplitude F_J-F_O
$W_{OI}=(F_t-F_O)/(F_I-F_O)$	Ratio of variable fluorescence F_t-F_O to the amplitude F_I-F_O
$\varphi P_t=PHI(P_t)=TR_t/ABS=1-F_t/F_M$	Quantum yield for primary photochemistry at any time t
$\varphi P_O=PHI(P_O)=TR_O/ABS=1-F_O/F_M$	Maximum quantum yield for primary photochemistry

$\psi E_O = PSI_O = ET_O/TR_O = (1-V_J)$	Probability that an electron moves further than Q_A^-
$\varphi E_O = PHI(E_O) = ET_O/ABS = (1-F_O/F_M)/(1-V_J)$	Quantum yield for electron transport (ET)
$\varphi D_O = PHI(D_O) = 1-\varphi P_O = F_O/F_M$	Quantum yield (at t = 0) of energy dissipation
$\varphi R_O = RE_O/ABS = \varphi P_O \times \psi E_O \times \delta R_O = \varphi P_O \times (1-V_I)$	Quantum yield for reduction of the end electron acceptors on the PSI acceptor side (RE)
$\delta R_O = RE_O/ET_O = (1-V_I)/(1-V_J)$	Probability that an electron is transported from the reduced intersystem electron acceptors to the final electron acceptors of PSI (RE)
$\gamma RC = CHlRC/CHltotal = RC/(ABS+RC)$	Probability that a PSII Chl molecule functions as RC
$ABS/CS = CHl/CS$	Absorption flux per Cs
$TR_O/CS = \varphi P_O \times (ABS/CS)$	Trapped energy flux per Cs
$RC/CS = \varphi P_O \times (VJ/M_O) \times (ABS/CS)$	Q_A^- reducing RCs per Cs
$PIABS = \frac{\gamma RC}{1-\gamma RC} \times \frac{\varphi Po}{1-\varphi Po} \times \frac{\psi Eo}{1-\psi Eo}$	Performance index (potential) for energy conservation from photons absorbed by PSII to the reduction of the intersystem electron acceptors

Subscript “o” indicates that the parameter refers to the onset of illumination, when all RCs are assumed to be open.

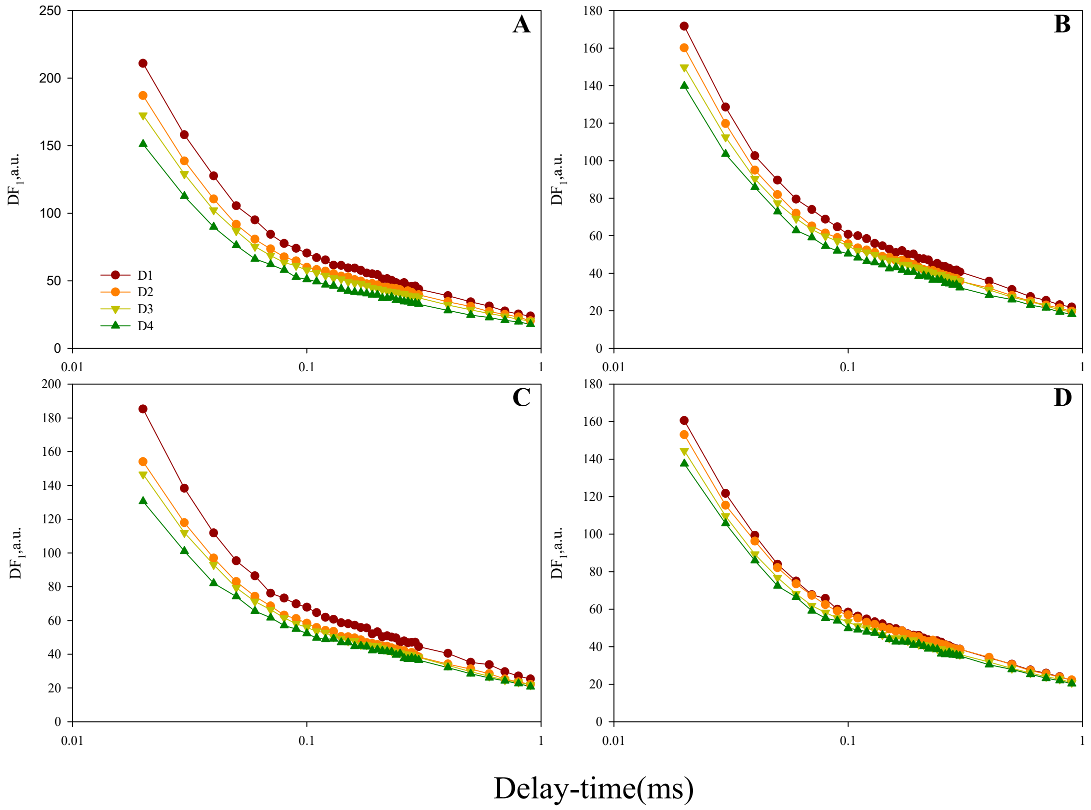

Figure S1. Kinetics of delayed fluorescence DF (in arbitrary units) at the characteristic steps I1 (7 ms JIP-time) of the two maize hybrids under different planting densities. A--B: 2019 data; C--D: 2020 data. (A, C): Absolute values of Zhengdan958. (C, D): Absolute values of Xianyu335. Signals are plotted on a logarithmic time scale.
